# ESM1 facilitates the EGFR/HER3-triggered epithelial-to-mesenchymal transition and progression of gastric cancer via modulating interplay between Akt and angiopoietin-2 signaling

**DOI:** 10.7150/ijbs.100276

**Published:** 2024-09-09

**Authors:** Yi-Chieh Yang, Ko-Hao Ho, Ke-Fan Pan, Kuo-Tai Hua, Min-Che Tung, Chia-Chi Ku, Ji-Qing Chen, Michael Hsiao, Chi-Long Chen, Wei-Jiunn Lee, Ming-Hsien Chien

**Affiliations:** 1 Department of Medical Research, Tungs' Taichung MetroHarbor Hospital, Taichung, Taiwan.; 2 Graduate Institute of Clinical Medicine, College of Medicine, Taipei Medical University, Taipei, Taiwan.; 3 Department of Medical Education and Research, Wan Fang Hospital, Taipei Medical University, Taipei, Taiwan.; 4 Division of Colorectal Surgery, Department of Surgery, Wan Fang Hospital, Taipei Medical University, Taipei, Taiwan.; 5 Graduate Institute of Toxicology, College of Medicine, National Taiwan University, Taipei, Taiwan.; 6 Department of Medical Research, China Medical University Hospital, China Medical University, Taichung, Taiwan.; 7 Department of Surgery, Tungs' Taichung Metro Harbor Hospital, Taichung, Taiwan.; 8 Department of Cancer Biology, Geisel School of Medicine at Dartmouth, Lebanon, NH, USA.; 9 Genomics Research Center, Academia Sinica, Taipei, Taiwan.; 10 Department of Pathology, Taipei Medical University Hospital and College of Medicine, Taipei Medical University Taipei, Taiwan.; 11 Department of Urology, School of Medicine, College of Medicine, Taipei Medical University, Taipei, Taiwan.; 12 TMU Research Center of Cancer Translational Medicine, Taipei Medical University, Taipei, Taiwan.; 13 Pulmonary Research Center, Wan Fang Hospital, Taipei Medical University, Taipei, Taiwan.; 14 Traditional Herbal Medicine Research Center, Taipei Medical University Hospital Taipei, Taiwan.

**Keywords:** ESM1, Patient-derived organoid, Gastric cancer, EGFR/HER3, Akt, Angiopoietin-2, EMT

## Abstract

Gastric cancer (GC) poses global challenges due to its difficult early diagnosis and drug resistance, necessitating the identification of early detection markers and understanding of oncogenic pathways for effective GC therapy. Endothelial cell-specific molecule 1 (ESM1), a secreted glycoprotein, is elevated in various cancers, but its role in GC remains controversial. In our study, ESM1 was elevated in GC tissues, and its concentration was correlated with progression and poorer patient prognosis in independent cohorts. Functionally, ESM1 expression promoted proliferation, anoikis resistance, and motility of GC cells, as well as tumor growth in PDOs and in GC xenograft models. Mechanistically, ESM1 expression triggered the epithelial-to-mesenchymal transition (EMT) of GC cells by enhancing epidermal growth factor receptor (EGFR)/human EGFR 3 (HER3) association and activating the EGFR/HER3-Akt pathway. Additionally, angiopoietin-2 (ANGPT2) was found to be highly correlated with ESM1 and interplayed with Akt to induce the EMT and cancer progression. Use of a signal peptide deletion mutant (ESM1-19del) showed that the secreted form of ESM1 is crucial for its protumorigenic effects by activating the EGFR/HER3-Akt/ANGPT2 pathway to promote the EMT. Patients with high levels of both ESM1 and ANGPT2 had the poorest prognoses. Furthermore, therapeutic peptides successfully inhibited ESM1's induction of the aforementioned signals and motility of GC cells. ESM1's oncogenic role in GC involves activating the EGFR/HER3-Akt/ANGPT2 pathway, presenting a potential therapeutic target for GC.

## Introduction

Globally, gastric cancer (GC) is the fifth most common type of cancer (over 1 million new cases), and the fourth leading cause of cancer-related deaths (over 0.7 million deaths) in 2020 1. The occurrence and development of GC are a multi-gene and multi-stage process, and the gradual accumulation of gene alterations leads to continued growth and metastatic advantage of neoplastic cells and substantially promotes the GC malignant progression 2, 3. Therefore, unlimited proliferation and metastasis are two major features of GC, and many patients die from cancer metastasis instead of the primary cancer 4. Because of the absence of major clinical manifestations, most GC patients are diagnosed at an advanced lymph node metastasis stage and are thus difficult to cure 5. Until now, novel therapies such as targeted therapy and immunotherapy have been established for treating advanced-stage GC. Unfortunately, not all patients respond to these treatments, and the mortality of advanced-stage GC remains stubbornly high 6. Thus, there is an urgent need to identify critical carcinogenic factors of GC, discover novel therapeutic targets, and develop effective targeted drugs for GC, to improve the clinical outcomes of advanced-stage GC patients.

The epithelial-to-mesenchymal transition (EMT) is an important step in the initiation and promotion of tumor proliferation, migration, and metastasis in GC 7. The EMT process was also reported to promote stemness, chemoresistance, and insensitivity to tyrosine kinase inhibitors (TKIs) in GC cells, and the EMT status is a critical prognosticator for GC 8, 9. Mechanistically, the epidermal growth factor (EGF) receptor (EGFR)-Akt, EGFR-signal transduction and activator of transcription 3 (STAT3), and mitogen-activated protein kinase (MAPK)/extracellular signal-regulated kinase (ERK) kinase (MEK)/ERK pathways are critical pathways responsible for the EMT progression triggered by several oncoproteins such as carcinoembryonic antigen-related adhesion molecule 6 10, EGF-like domain 7 11, aquaporin 3 12, bone morphogenetic protein-2 13, and hypoxia-inducible factor-1α 14. Subsequently, the EMT in GC can be modulated by various transcriptional factors (Snail, Slug, Twist, Zeb1 and so on) which are downstream of the above-mentioned signaling pathways 7.

Endothelial cell-specific molecule 1 (ESM1), also called endocan, is a secreted dermatan proteoglycan harboring 165 amino acids (aas) and a single mucopolysaccharide chain (dermatan sulfate (DS) chain) that is covalently linked to the 137th serine residue. Originally, ESM1 was identified in endothelial cells as playing a vital role in regulating endothelial cell functions, such as angiogenesis 15. Recently, accumulating reports have demonstrated that ESM1 is overexpressed in various tumor types including lung, prostate, head and neck, kidney, and other cancers 16. Upregulation of ESM1 in these cancers was shown to modulate different aspects of tumor progression, such as enhancing cell mobility, proliferation, stemness, and oncogenic pathway activation 16. Mechanistic studies showed that activation of the Akt-dependent nuclear factor (NF)-κB/cyclin D1 pathway is critical for ESM1-modulated proliferation of multiple types of cancer 16. Our previous study showed that ESM1 can promote EGFR-driven lung tumor proliferation 17. Moreover, ESM1 was also reported to be a potential marker of the tumor EMT and metastasis in colorectal cancer 18. Furthermore, we recently showed that ESM1 maintains cancer stemness and promotes metastasis of prostate cancer via coordinating the Wnt/β-catenin pathway 19. As to the role of ESM1 in GC, upregulation of ESM1 was also observed in GC tissues and intratumoral vessels, and was reported to be a poor prognostic factor 20. In contrast, some studies observed that ESM1 levels are positively correlated with differentiation levels and that ESM1 overexpression can induce cell apoptosis and reduce migration 21, 22. However, the experimental conditions of those studies were limited to a single cell line, thus leaving the conclusion open to debate. While controversial roles of ESM1 in promoting GC were established in recent years, the precise molecular mechanism through which ESM1 is involved in GC are still unclear.

In the present study, we showed that ESM1 is a poor prognostic factor, and its overexpression was associated with advanced clinical stage development, vascular invasion, and lymph node metastasis in GC patients. Moreover, overexpression of ESM1 promoted EMT progression, colony formation, migration/invasion, and anoikis resistance in GC cells, and we further demonstrated that secretion of ESM1 is essential for ESM1-modulated GC progression. Mechanistically, we found that ESM1 facilitated the interaction of EGFR/human EGFR 3 (HER3) and activated the EGFR/HER3-Akt pathway to trigger EMT progression in GC. In addition, angiopoietin-2 (ANGPT2) was highly correlated with ESM1 and interplayed with Akt to induce the EMT and cancer progression in GC. Our study highlights the significance of ESM1 in GC and the potential of targeting ESM1-EGFR/HER3 regulation in therapeutic applications against GC.

## Materials and methods

### *In silico* bioinformatics analysis

Gene expression levels of ESM1 were retrieved from the GSE27342, GSE66229, and GSE13861 microarray datasets of the Gene Expression Omnibus (GEO) database; these contain transcriptome profiles of normal, adjacent non-tumor, or GC tumor tissues, and related clinicopathological parameters 23-25. The 2856995, 208394_x_at, and ILMN_1773262 ESM1 probes were respectively used for the GSE27342, GSE66229, and GSE13861 analyses. 205572_at and 211148_s_at were probes for ANGPT2 expression. Box plots for ESM1 expression values were created with respect to the clinical stage lymph node metastasis, and vascular invasion status. The prognostic significance of *ESM1* and *ANGPT2* levels and the combined effects of both genes in patients with GC were determined using a Kaplan-Meier (KM) analysis. ESM1 levels and survival data of GC subjects were also obtained from the KM Plotter (https://kmplot.com/analysis/). The STRING website (https://string-db.org/) was utilized to explore protein-protein interaction (PPI) networks of ESM1-regulated proteins. Further, survival mapping of selected genes in different cancer types was carried out by specific tools available at the GEPIA2 database (http://gepia2.cancer-pku.cn/#index).

### Cell lines and cell culture

Human AGS, KATO-III, and N87 GC cells were obtained from American Type Culture Collection (ATCC; Manassas, VA, USA). MKN45 cells were purchased from the Health Science Research Resources Bank (Osaka, Japan). All cell lines were maintained in RPMI 1640 medium (Gibco-BRL, Gaithersburg, MD, USA) supplemented with 1% penicillin-streptomycin-glutamine (Sigma-Aldrich, St. Louis, MO, USA) and 10% fetal bovine serum (FBS) (Gibco-BRL) and grown as adherent monolayer cultures at 37 °C with 5% CO_2_ in a humidified incubator.

### Preparation of total cell extracts and Western blot analysis

The procedures for total protein lysate extraction and Western blot analysis were as described previously 26. Briefly, each sample with 30 to 40 µg of protein was separated by SDS-PAGE and transferred to PVDF membranes. After blocking with 5% skim milk in phosphate-buffered saline (PBS) with 0.05% Tween 20 (PBST), membranes were incubated with primary antibodies at 4 °C overnight. Next, membranes were incubated with appropriate secondary antibodies conjugated with horseradish peroxidase (HRP) at room temperature for 1 h and developed with enhanced chemiluminescence (ECL) reagents (Millipore, Billerica, MA, USA) to detect expression signals. The following primary antibodies were used: endocan (LIA-1001; Lunginnov, Lille, France); phosphorylated (p)-EGFR (#3777), p-Akt (#9271), p-ERK (#4370), p-STAT3 (#9145), EGFR (#2239), Akt (#9272), STAT3 (#9139), ERK (#4695), E-cadherin (#3195), vimentin (#5741), Snail (#3879), Slug (#9585), p-HER3 (#2842), and HER3 (#12708) antibodies all obtained from Cell Signaling Technology (Danvers, MA, USA); angiopoietin-2 (bs-0677R-TR) and p-HER3 (bs-3491R) antibodies purchased from Bioss (Woburn, MA, USA); α-tubulin (#66031-1-Ig) antibodies respectively obtained from Proteintech (Rosemont, IL, USA).

### Dot blot assay

Briefly, 10^6^ GC cells were stably infected with a virus carrying either ESM1, ESM1-19del, ESM1-shRNA, or their respective controls and seeded in 6-cm Petri dishes for 16 h. Next, the cell culture medium was replaced by complete medium for a further 24 h and harvested for dot blotting. Supernatants were blotted onto nitrocellulose membranes via a 96-well dot-blotter (GFE9600; BIOMAN Scientific, New Taipei City, Taiwan). Hybridization and detection of each dot were performed according to the Western blot protocol.

### Cell proliferation and colony-formation assays

GC cells expressing ESM1, shESM1, or a control vector were seeded in 96-well plates (at 5×10^3^ cells/well) containing culture medium with 10% FBS for the indicated times and then subjected to a cell proliferation assay (Cell Counting Kit (CCK)-8 assay; Sigma-Aldrich, St. Louis, MO, USA) according to the manufacturer's instructions. Data were collected from three replicates. For the colony-formation assay, ESM1-overexpressing or -knockdown (KD) GC cells were seeded in six-well dishes (at 10^3^ cells/well) and then cultured under standard conditions. The medium was changed every 2 days, and after 7~10 days of incubation, cells were stained with crystal violet (1%), and colonies were manually counted using ImageJ software (National Institutes of Health, Bethesda, MD, USA).

### Anoikis-resistant cell-survival rate assay

GC cells expressing ESM1 or a control vector were seeded in ultra-low attachment 24-well plates (Corning Costar, Cambridge, MA, USA) at a density of 1500 cells/well. Suspended cells were incubated under normal culture conditions at 37 °C for 48 h and collected for the CCK-8 assay to detect the survival rate.

### Transwell-migration and -invasion assays

Transwell-migration and -invasion assays were performed according to our previous study 27 to evaluate the migratory and invasive abilities of GC cells which overexpressed ESM1, mutant ESM1, or shESM1, or had been treated with therapeutic peptides. Briefly, ESM1-manipulated GC cells were seeded in a noncoated top chamber for the migration assay or a Matrigel (BD Biosciences, Bedford, MA, USA)-coated top chamber for the invasion assay. The top chamber contained serum-free medium, while the lower chamber contained complete medium as a chemoattractant for cells in the top chamber. Cells were allowed to migrate through or invade the membrane for 24 or 48 h, after which the migrated and invaded cells were fixed and stained with crystal violet (0.5%). The number of migrated and invaded cells was counted in at least three random microscopic fields (×100 or ×200).

### Immunofluorescence (IF) analysis

IF techniques were used to observe the actin rearrangement after overexpressing ESM1 in GC cells. Briefly, GC cells were seeded on coverslips overnight and fixed by adding 4% paraformaldehyde and incubating for 30 min at room temperature. After washing cells with PBS, fixed cells were permeabilized with 0.1% Triton X-100 for 1 h and stained with Alexa Fluor 594 phalloidin (ThermoFisher Scientific, Rockford, IL, USA) at room temperature in the dark for another 1 h. After washing cells with PBS, a coverslip was mounted onto a glass slide using mounting medium with DAPI, and slides were further examined and photographed using a Zeiss Axiophot fluorescence microscope (Carl Zeiss Microimaging, Gottingen, Germany).

### RNA preparation and reverse-transcriptase (RT)-PCR

Messenger (m)RNA was prepared and amplified according to our previous study. Primer sequences of ANGPT2 were F: 5'-ACCCCACTGTTGCTAAAGAAGA-3' and R: 5'-CCATCCTCACGTCGCTGAATA-3'.

### 3D organoid culture

Patient-derived organoids (PDOs) from metastatic gastroesophageal cancer patients 28, which were kindly provided by Dr. Kelvin Kun-Chih Tsai (Taipei Medical University). About 50 μl of Matrigel/well was used for coating as a basic layer on six-well plates; (2.5~3) × 10^5^ cells/well were gently mixed with 200 μl Matrigel and seeded above on the basic layer. The base culture medium for PDOs maintaining was Dulbecco's modified Eagle medium (DMEM)/F12 supplemented with B27, N2 additive, bovine serum albumin (BSA; 0.01%), L-glutamine (2 mM), EGF (50 ng/ml), Noggin (100 ng/ml), R-Spondin 1 (500 ng/ml), gastrin (10 nM), fibroblast growth factor (FGF)-10 (10 ng/ml), FGF-basic (10 ng/ml), Wnt-3A (100 ng/ml), prostaglandin E_2_ (1 μM), Y-27632 (10 μM), nicotinamide (4 mM), A83-01 (0.5 μM), SB202190 (5 μM), and 100 units/mL penicillin-streptomycin. Organoid cells were maintained at 37 °C in a 5% CO_2_ humidified atmosphere, and the medium was changed every 3~4 days for 10-14 days.

### DNA construct establishment and lentiviral production and infection

An ESM1-overexpressing construct was engineered by a polymerase chain reaction (PCR) and subcloned into a pWPI vector. Short hairpin (sh)RNA constructs against ESM1 and ANGPT2 were purchased from the RNA Technology Platform and Gene Manipulation Core (Taipei, Taiwan). Target sequences were: sh-ESM1-1: TGG CAT CTG GAG ATG GCA ATA, and sh-ESM1-2: CTG AGG TGT CAG CCT TCT AAT, shANGPT2: GCT TAC TCA TTG TAT GAA CAT. Lentiviral particles were produced based on a previously described protocol 29. Briefly, 293T packaging cells were transfected with 10 μg of the pWPI-ESM1- or shESM1- and shANGPT2-expressing plasmids together with 10 μg pCMVDR8.91 (the packaging vector) and 1 μg pMD.G (the enveloping vector). After 16 h of incubation, the transfection medium was replaced with fresh culture medium for a subsequent 48 h, and lentivirus-containing medium was further collected by centrifugation at 1500 rpm. Next, GC cells were infected with fresh lentivirus-containing medium (supplemented with 8 μg/ml polybrene) for 24 h and subjected to different functional assays.

### GC patients of a tissue microarray (TMA)

GC patient tissues, along with their corresponding paired normal tissues, were procured from Taipei Municipal Wan Fang Hospital in Taiwan between 1998 and 2011 for this study. Follow-up data were recorded until April 2014. Clinical and pathological information of patients in this cohort was retrospectively collected from medical records. The tumor, lymph node, and metastatic (TNM) stages of GC patients were classified according to the 7th edition of the Cancer Staging Manual by the American Joint Committee on Cancer. Pathologist C.L. Chen confirmed the adequacy of all samples in the TMA. The study was conducted with approval (no. 99049 in 2010) from the Institutional Review Board of Wan Fang Hospital and permission from the ethics committees of the institution involved.

### IHC staining and interpretation

All tumor tissue samples were fixed in a 10% buffered formaldehyde solution. Specimens were embedded in paraffin blocks, and 4-µm sections were cut. The IHC staining processes were previously described 30. In brief, paraffin-embedded tumor tissues were deparaffinized with xylene and incubated in sodium citrate buffer (10 mM sodium citrate and 0.05% Tween 20, at pH 6.0) in a steam oven to enhance antigen retrieval. After blocking endogenous peroxidase activity with 0.3% H_2_O_2_, slides were washed with PBS and further blocked with 5% FBS in PBS for 1 h at room temperature. Slides were then incubated with anti-ESM1 (H00011082-M02), anti-ANGPT2 (bs-0677R), anti-Snail + Slug (ab85936), and anti-Ki67 (9027S) antibodies overnight at 4 ℃. After washing in PBS, slides were developed with a Novolink Polymer Detection Systems kit (Leica Biosystems, Deer Park, IL, USA) according to the manufacturer's instructions. Hematoxylin staining of specimens for 1 min was used as a light counterstain. To interpret IHC results, the staining intensity was graded from 0 to 3 (respectively representing no, weak, moderate, and strong staining). Additionally, the percentage of positively stained cells (ranging from 0% to 100%) was documented. Final IHC scores were calculated by multiplying the staining intensity by the percentage of positive cells, resulting in a range of 0 to 300.

### Lentivirus-based gene delivery for PDOs

A 12-well plate was first coated with 80 μL Matrigel. A single cell suspension with (1~1.5) × 10^5^ cells in 500 μL of organoid culture medium was added with an optimal volume of viral particles in a 1.5-mL microtube. About 2.5 μL of TransDux™ (System Biosciences, Palo Alto, CA, USA) was added to the microtube and gently mixed. The 500 μL total mixture with a single-cell suspension and viral particles was transferred onto solidified Matrigel, and incubated at 37 ℃ overnight. Then the medium was removed after at least 16 h, and cells were covered with 60 μL Matrigel. About 800 μL of organoid culture medium was added to each well and refreshed every 3~4 days.

### Tumor xenograft model

Luciferase-tagged NCI-N87 cells (10^6^) expressing ESM1-HA, 19del-ESM1, or a control vector were suspended in PBS, and these different NCI-N87 cells were subcutaneously implanted into 6-8-week-old nonobese diabetic (NOD)-SCID mice. All mice with an abdominal incision received the same anesthetic regimen. To follow tumor growth in the mice, the tumor volume was measured weekly and calculated as 1/2 × length × width^2^ (in mm^3^). After 28 days, NCI-N87-injected mice were sacrificed, and tumor specimens were further harvested, photographed, sectioned, and stained with hematoxylin and eosin (H&E) for further histopathological analyses. All animal experiments were performed under a protocol approved by the Institutional Animal Care and Use Committee of Taipei Medical University (LAC-2023-0351).

### Statistical analysis

*In vitro* cell line experiments were performed thrice, and experimental data are presented as the mean ± standard deviation (SD). The KM curve showed the cumulative survival analysis and was conducted by Statistical Product and Service Solutions software (SPSS), vers. 20 (IBM, Endicott, NY, USA). Differences between two groups were analyzed using Student's *t*-test in GraphPad Prism 5 (GraphPad Software, San Diego, CA, USA). Correlations of ESM1 with ANGPT2 and clinicopathologic parameters of GC were examined by Pearson's Chi-squared test. *p* < 0.05 was considered a statistically significant difference.

## Results

### ESM1 is elevated in GC and correlated with poor clinical outcomes

**Figure 1:**
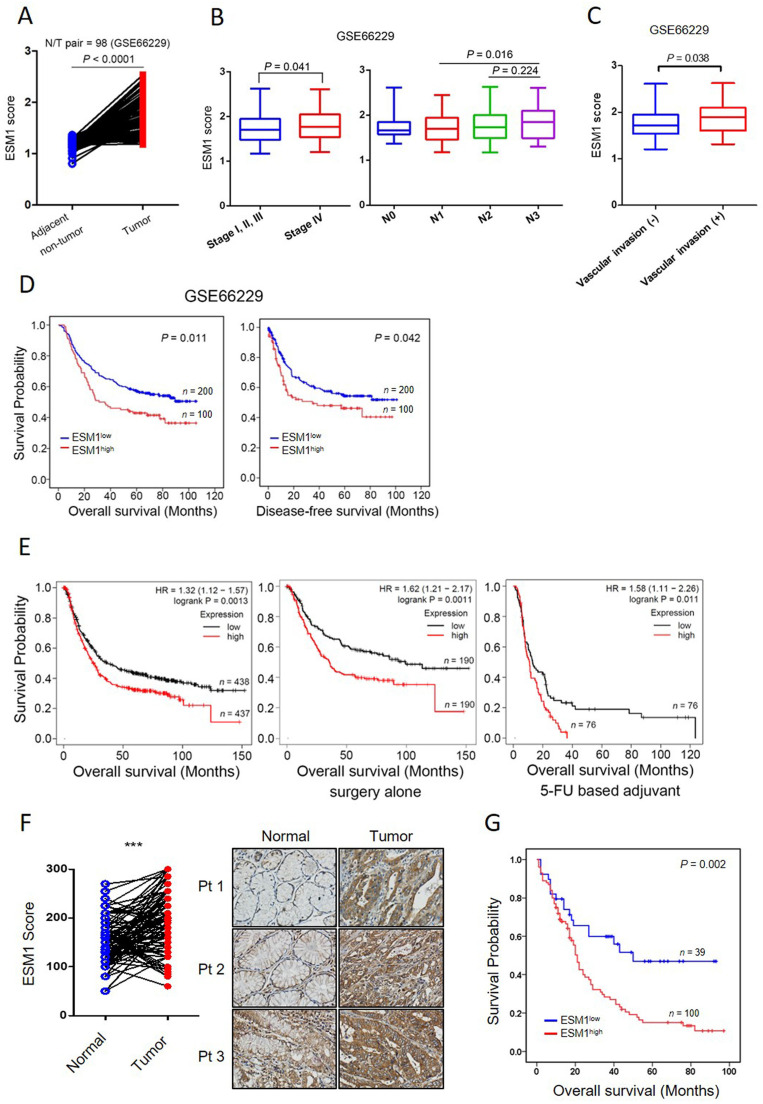
**ESM1 is highly expressed in gastric cancer (GC) and associated with a poor prognosis.** (**A**) Gene expression levels of ESM1 in paired adjacent (GSE66229) normal and tumor tissues derived from patients with GC. Statistical significance was analyzed by a paired *t*-test. (**B, C**) *ESM1* gene expression levels in GC from the GSE66229 dataset were compared according to clinical stages and the lymph node metastasis (**B**), and vascular invasion (**C**) statuses. (**D**) Kaplan-Meier (KM) survival analysis showing the correlation between ESM1 expression and the overall survival (OS) or disease-free survival of GC patients based on the GSE66229 dataset. (**E**) Correlation between ESM1 expression and OS in GC patients receiving different treatments as determined using a KM plotter database. Gene expression was dichotomized into high and low values using the median as a cutoff. HR, hazard ratio. (**F**) Representative pictures and quantified results of IHC staining of ESM1 levels in normal and tumor tissues. (**G**) KM survival analysis showing the correlation between ESM1 expression and the OS of GC patients based on Taiwanese cohorts.

We initially elucidated the clinical relevance of ESM1 in GC using GEO databases. Analysis of a public microarray dataset (GSE27342), comprising 80 GC tumor tissues and their corresponding adjacent normal tissues, revealed significant upregulation of ESM1 in GC tissues (Figure S1A). This observation was corroborated by an analysis of two additional microarray datasets: one containing 98 GC N (normal)/T (tumor) paired cohorts (GSE66229) (Figure 1A) and another comprising 71 GC tissues with 19 normal tissues (GSE13861) (Figure S1B). Subsequently, we further analyzed correlations of ESM1 expression with patients' clinicopathological features and survival rates. In datasets GSE13861 and GSE66229, significantly higher levels of ESM1 transcripts were observed in GC patients with advanced clinical stages (stage 4) and lymph node metastasis (N3) compared to patients with earlier clinical and N stages (Figures 1B, S1C). In the GSE66229 dataset, elevated ESM1 transcripts were also found in GC patients with vascular invasion (Figure 1C). Moreover, Chi-square tests also validated that high ESM1 expression was correlated with advanced N stages and vascular invasion in GC patients from the GSE66229 dataset (Table S1). KM plot analyses from the same dataset revealed that GC patients with high ESM1 tumor expression had shorter overall survival (OS) (*p* = 0.011) and disease-free survival (DFS) (*p* = 0.042) times compared to those with low ESM1 tumor expression (Figure 1D).

Furthermore, univariate and multivariate analyses of OS indicated that the ESM1 expression level could serve as an independent predictor of survival for GC patients (Table S2). Consistent with results from the individual GEO databases, analysis of the GC cohort from KM plotter databases also revealed a positive correlation between ESM1 expression and poor OS (*p* = 0.0013), regardless of whether GC patients had only received surgery (*p* = 0.0011) or also chemotherapy (*p* = 0.011) (Figure 1E). In addition to the analysis of ESM1 transcript levels, we also observed higher ESM1 protein levels in GC tissues than in non-cancerous parts in our Taiwanese cohort (*n*=93; *p* < 0.0001) (Figure 1F). Importantly, higher ESM1 protein levels were also positively correlated with poorer prognostic outcomes (*p* = 0.002) (Figure 1G). Taken together, these data indicated that high ESM1 expression may play a critical role in GC progression.

### ESM1 expression enhances the malignant properties of GC cell lines and the growth of patient-derived organoids (PDOs)

**Figure 2:**
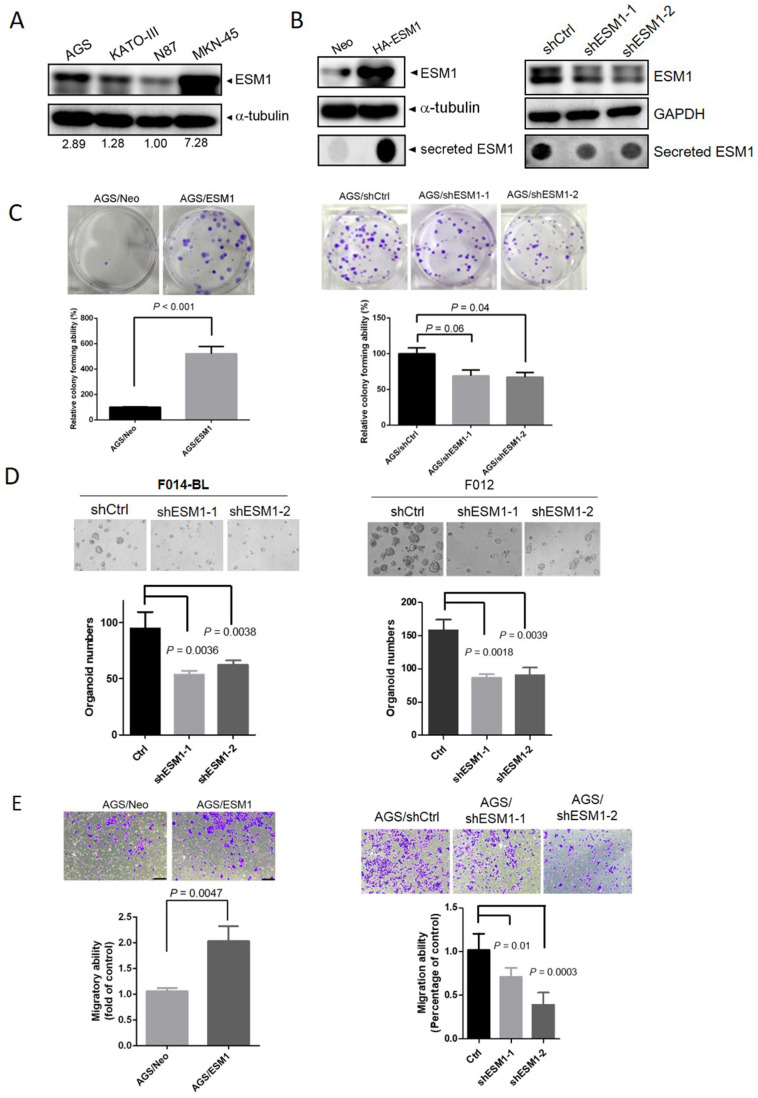
**ESM1 expression promotes the growth and motility of gastric cancer (GC) cells.** (**A**) Protein expression levels of ESM1 in GC (AGS, KATO-III, N87, and MKN-45) cell lines. Quantitative results of ESM1 proteins were adjusted to α-tubulin protein levels. (**B**) Western blot and dot blot analyses respectively revealed endogenous and secreted ESM1 levels in AGS cells expressing ESM1-HA (left panel) or ESM1 shRNA (right panel). (**C**) Colony-forming abilities of ESM1-manipulated AGS cells. The quantified data were examined by calculating colony numbers per well. (**D**) The growth number of 3D patient-derived organoids (PDOs) was measured after knockdown of ESM1 in F014-BL and F012 cells. Levels of magnification of the representative images were 10x. (**E**) Migratory ability of ESM1-manipulated AGS cells was determined by a transwell migration assay. Data in **C-E** are presented as the mean ± SD.

To further elucidate the oncogenic functions of ESM1 in GC, we initially assessed endogenous levels of ESM1 in a panel of GC cell lines, including poorly differentiated (AGS, KATO-III, and MKN-45) and well-differentiated (N87) cells, using Western blotting. We found that poorly differentiated GC cells expressed higher ESM1 levels than did well-differentiated GC cells (Figure 2A). Subsequently, AGS cells were transfected with lentiviral vectors containing shRNA targeting ESM1 (sh-ESM1) or ESM1-overexpressing plasmids (HA-ESM1), with the efficiency confirmed by Western blot and dot blot analyses (Figure 2B). Proliferation rates derived from the CCK-8 assay indicated that ESM1-KD significantly reduced the growth of AGS cells, while cell proliferation was not significantly affected by ESM1 overexpression (Figure S2A, B). Moreover, results from the plate clonogenic assay demonstrated that colony-forming abilities of AGS cells were suppressed by ESM1 depletion (Figure 2C; right panel), but dramatically increased by ESM1 overexpression (Figure 2C; left panel). Similarly, overexpression and knockdown of ESM1 respectively promoted and attenuated colony-forming abilities of KATO-III (Figure S2C; left panel) and MKN-45 cells (Figure S2C; right panel). Importantly, we also observed that ESM1 depletion significantly reduced the growth of PDOs, F014-BL and F012 (Figure 2D), obtained from metastatic gastroesophageal cancer patients 28. A notable reduction in the tumor growth ability was observed in both 2D culture conditions and the 3D PDO system.

In addition to its effect on the growth of GC cells, we further observed that ESM1 overexpression enhanced the survival rate of AGS cells grown in suspension, known as anoikis resistance (Figure S2D). Anoikis resistance was identified as a prerequisite for metastasis 31. Therefore, we conducted further evaluations to assess ESM1's role in modulating cell migration and invasion, two fundamental steps of tumor metastasis. Using a transwell assay, we observed increases in the numbers of migratory and invasive cells upon ESM1 overexpression in AGS cells (Figure 2E; left panel, Figure S2E). Conversely, ESM1-KD in AGS cells led to a decrease in the migratory ability (Figure 2E; right panel). Collectively, these results emphasize the oncogenic roles of ESM1 in GC progression.

### ESM1 promotes the EMT via activating the EGFR-regulated signaling pathway in GC

**Figure 3:**
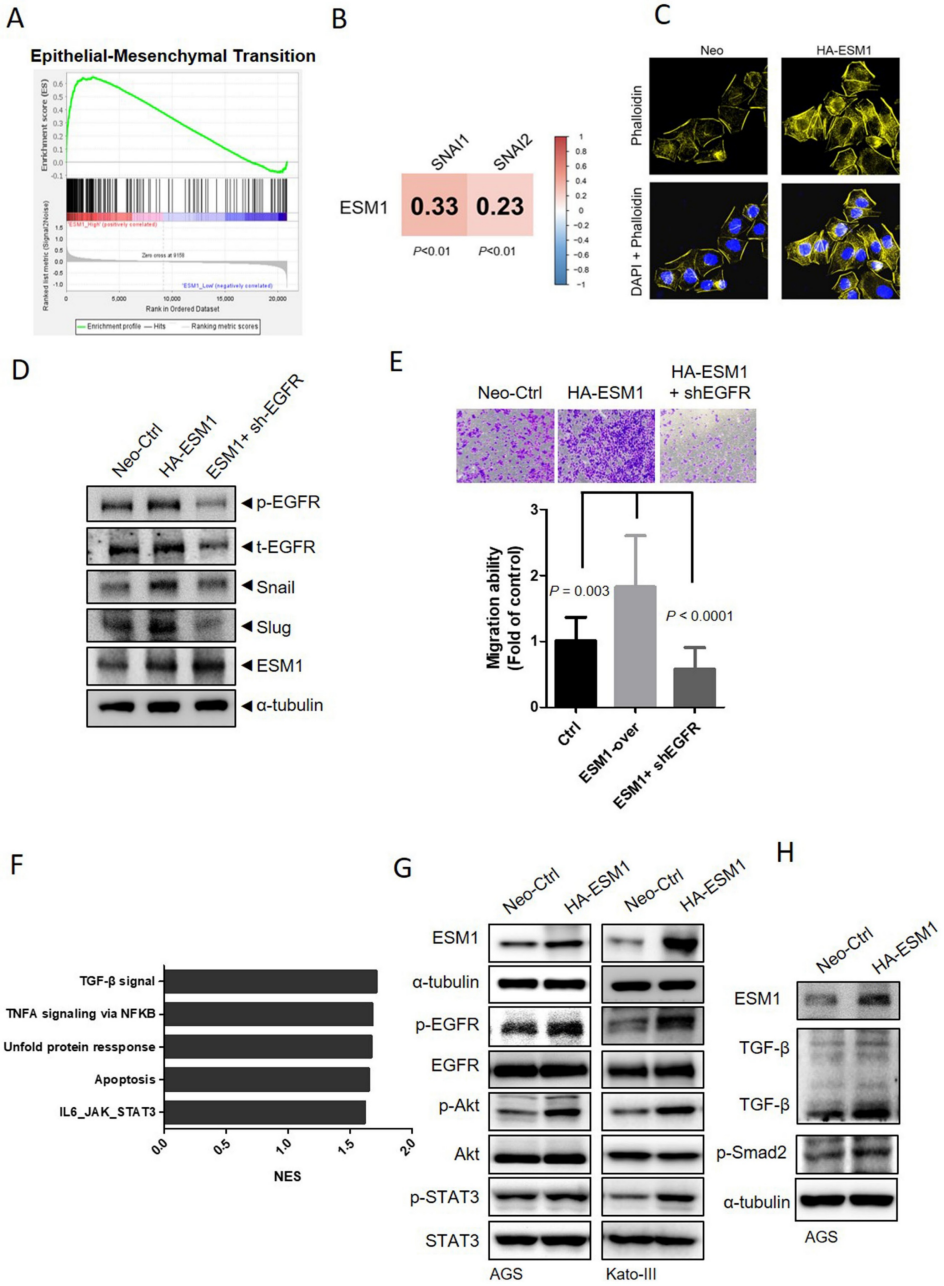
** ESM1 induces the epithelial-to-mesenchymal transition (EMT) of gastric cancer (GC) cells through activating epidermal growth factor receptor (EGFR)-dependent signaling.** (**A**) Gene set enrichment of the EMT in GC patients with high (top 5%) versus low (bottom 5%) expression of ESM1. An EMT gene set derived from HALLMARK was used. (**B**) Positive correlations of ESM1 and the mesenchymal markers, Snail and Slug, in 300 GC patients. (**C**) Cellular microfilament bundle rearrangements were induced by ESM1 overexpression in AGS cells. Cells that overexpressed ESM1-HA or a control vector (Neo) were seeded on coverslips overnight. Cells were then fixed and stained for F-actin by Alexa Fluor 594 phalloidin. Nuclei were counterstained with DAPI (blue). Original magnification, 400×. (**D**) Western blot and migration analyses to examine levels of phosphorylated (p)-EGFR, t-EGFR, ESM1, Snail, and Slug under manipulation of ESM1 and EGFR levels. (**E**) The migratory ability of ESM1/EGFR-manipulated AGS cells. (**F**) The top five enriched pathways of GC patients (GSE66229) with high (top 5%) versus low (bottom 5%) expressions of ESM1 obtained by a GSEA. (**G**) Detection of activation of the EGFR (pTyr1068) and its downstream signaling including Akt (pSer473) and signal transduction and activator of transcription 3 (STAT3) (pTyr705) by Western blotting after overexpression of ESM1 in AGS and Kato-III cells. (**H**) Examining activation of transforming growth factor (TGF)-β and p-Smad2 signals in ESM1-overexpressing AGS cells.

The initiation and promotion of tumor proliferation, migration, and metastasis in GC are facilitated by the EMT, a crucial step 7. To further explore the correlation of ESM1 and the EMT in GC, we obtained transcriptomic data from GC patients, which were retrieved from GSE66229, and examined differences in gene profiles between the two groups with the highest and lowest 5% ESM1 expression levels (Figure S3A). Through a gene set enrichment analysis (GSEA), enrichment of the EMT signature in ESM1-high patients was observed (Figure 3A). Specially, positive correlations of ESM1 with the EMT-related transcription factors Snail and Slug were also observed in GC patients (Figure 3B). Dynamic actin cytoskeletal remodeling plays an important role in EMT progression 32. We next determined whether ESM1 promotes cell motility accompanied by changes in the actin cytoskeleton. Alexa Fluor 594 phalloidin and DAPI were used to respectively stain actin filaments and nuclei, and results revealed that AGS cells overexpressing ESM1 contained more microfilament bundles than did parental cells (Figure 3C), while downregulation of ESM1 caused an opposite effect (Figure S3B), suggesting that dynamic actin reorganization may be involved in ESM1-modulated cell motility of GC cells. Additionally, activation of EGFR/PI3K/Akt was reported to be a common regulator for EMT-like transformation and anoikis resistance in cancers 33, 34. In our previous study, we demonstrated ESM1's importance in activating the EGFR in lung cancer 17. In this study, we also observed a significant upregulation in EGFR phosphorylation levels accompanied by induction of Snail and Slug upon ESM1 overexpression in GC cells.

Intriguingly, EGFR-KD effectively negated ESM1-induced expressions of Snail and Slug (Figure 3D). The observed protein expression levels were consistent with the migratory abilities of AGS cells, which were significantly abolished by EGFR depletion (Figure 3E). Taken together, these data suggest that ESM1-driven EGFR activation may play a role in promoting the EMT and GC progression. To further examine the yet-unexplored mechanism of ESM1 in GC, a GSEA based on the GSE66229 dataset was performed to identify the top five Hallmark gene sets among the ESM1-high group, including the TGF-β and interleukin (IL)-6/Janus kinase (JAK)-STAT3 signaling pathways which are associated with cell survival and mobility (Figure 3F). Despite some conflicting functions of TGF-β signaling in different stages of cancer progression 35, previous studies indicated that EGFR-Akt signaling switches TGF-β's function in breast cancer cells from antiproliferation to cancer promotion via EMT induction 36. Meanwhile, increased activation of the EGFR was shown to sustain STAT3 activation in response to IL-6 stimulation in colon cancer and lung adenocarcinoma models 37, 38. We found that overexpression of ESM1 increased activation of EGFR-driven signaling pathways, including Akt and STAT3, in AGS, Kato-III, and NCI-N87 cells (Figures 3G, S3C), while ESM1-KD caused opposite effects in AGS cells (Figure S3D). Notably, levels of TGF-β and its downstream signal, p-Smad2, increased upon ESM1 overexpression in GC cells (Figure 3H), but ESM1-KD led to opposite results (Figure S3E). All of these results hinted that Akt, STAT3, and TGF-β may be EGFR-modulated signaling pathways that participate in ESM1-driven EMT oncogenic properties in GC.

### ANGPT-2 highly correlates with ESM1 and interplays with Akt to promote the EMT and GC progression

**Figure 4:**
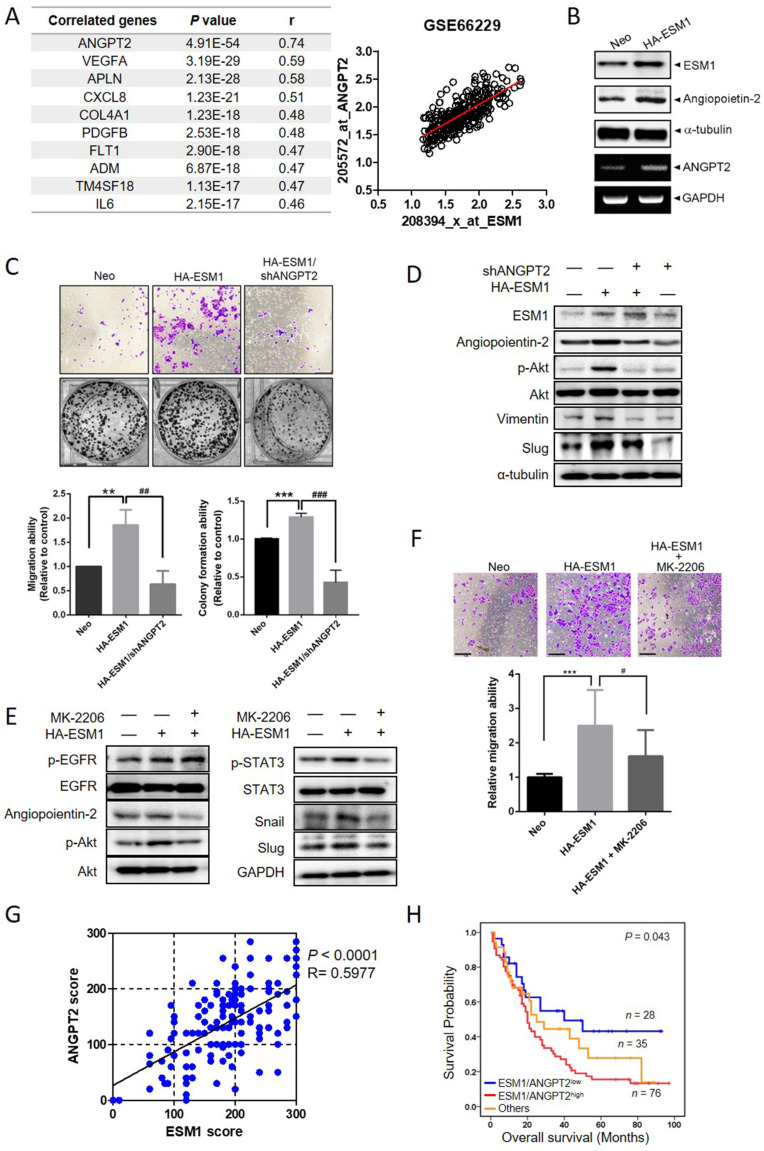
**Angiopoietin-2 (ANGPT2) is highly correlated with ESM1 and interplays with Akt to promote the epithelial-to-mesenchymal transition (EMT) and progression of gastric cancer (GC) cells.** (**A**) List of the top 10 ESM1-correlated genes with the highest Pearson's correlation coefficient obtained from the GSE66229 database (left panel). Visualized dot plot diagram of a correlation between ANGPT2 and ESM1 in 300 GC patients (right panel). (**B**) AGS cells overexpressing ESM1-HA or a control vector as indicated. Cell protein and RNA were extracted, and ESM1 and ANGPT2 expression levels were determined by Western blotting (upper panel) and RT-PCR (lower panel), respectively. (**C-D**) AGS cells overexpressing ESM1-HA, ESM1-HA+shANGPT2, shANGPT2, or a control vector as indicated were subjected to transwell-migration and colony-formation assays (**C**), and also a Western blot analysis (**D**). Multiples of differences are presented as the mean ± SD. ** *p* < 0.01, *** *p* < 0.001, compared to the control group; ^##^ *p* < 0.01, ^###^ *p* < 0.001, compared to the ESM1-overexpressing only group. (**E, F**) AGS/ESM1-HA cells were treated with 1 μM MK-2206 or vehicle for 24 h and the expression of indicated targets was determined by Western blotting (**E**), and the migration ability was further examined (**F**). (**G**) Positive correlation of protein levels of ESM1 and ANGPT2 in Taiwanese GC patients. (**H**) Kaplan-Meier curves of overall GC patient survival, grouped by ESM1 and ANGPT2 expressions. The *p* value indicates a comparison between patients with ESM1^high^/ANGPT2^high^, ESM1^low^/ANGPT2^low^, and others. The GC dataset was retrieved from a Taiwanese GC patient cohort.

To further explore potential ESM1-correlated genes, we analyzed 300 human GC samples retrieved from the GSE66229 dataset and found that the *ANGPT2* gene exhibited the highest correlation with ESM1 in GC tissues, with a Pearson correlation coefficient (*r*) of 0.74 (Figure 4A, *p* = 4.91E-54). This finding was consistent in another 440 GC cohort from The Cancer Genome Atlas (TCGA) database (Figure S4A). From the STRING database, we found that protein-protein interaction networks for ESM1 and ANGPT2 had a high confidence score (0.568; Figure S4B). In AGS and Kato-III cells, ESM1 overexpression actually promoted upregulation of ANGPT2 protein and messenger (m)RNA levels (Figures 4B, S4C). Functionally, ANGPT2-KD significantly reversed the enhanced migratory and colony-forming abilities induced by ESM1 overexpression in AGS cells (Figure 4C). Mechanistically, upregulation of ANGPT2 caused by ESM1 overexpression was significantly abolished by EGFR depletion (Figure S4D), suggesting that ANGPT2 is a downstream regulator of the ESM1-EGFR axis. We attempted to elucidate interactions among ANGPT2, Akt, and STAT3 signals involved in the EGFR-promoted EMT caused by ESM1 overexpression. ANGPT2-KD rescued ESM1 overexpression-induced increases of p-Akt, vimentin, and Slug (Figure 4D). Conversely, treatment with the Akt-specific inhibitor, MK-2206, markedly reversed ESM1-induced increases in ANGPT2, p-Akt, p-STAT3, Snail, and Slug (Figure 4E), as well as the migratory ability (Figure 4F) and anoikis resistance (Figure S4E) in GC cells. These results collectively indicated that ANGPT2 may play a crucial role in the ESM1-EGFR-Akt-STAT3 axis-induced EMT in GC cells through a regulatory network between Akt activation and GC progression. In the clinic, we also observed significant positive correlations between protein levels of ESM1 and ANGPT2 in GC patients via IHC staining (Pearson *r* = 0.5977; *p* < 0.0001) (Figure 4G and S4F), which were similar to mRNA expression observed in online databases. Additionally, ANGPT2 expression in GC was significantly correlated with expressions of the mesenchymal markers, vimentin (VIM), Snail (SNAI1), and Slug (SNAI2), in the same aforementioned GSE66229 dataset (Figure S4G). To further investigate the prognostic role of ANGPT2 and mesenchymal markers, we performed a survival analysis using the GEPIA2 database. Among 33 different types of cancer, we found that high ANGPT2 and mesenchymal marker expressions all had poor prognostic impacts on three cancer types including GC (Figure S4H). Moreover, GC patients with ESM1^high^/ANGPT2^high^ tumors had shorter survival times compared to those with ESM1^low^/ANGPT2^low^ tumors in Taiwanese, GSE66229, and TCGA cohorts (Figures 4H, S4I). Collectively, these clinical data implied that the ESM1-induced ANGPT2-mediated EMT was associated with a poor prognosis in GC patients.

### The secretory form of ESM1 is crucial for its oncogenic role in GC cells, PDOs, and a xenograft model

**Figure 5:**
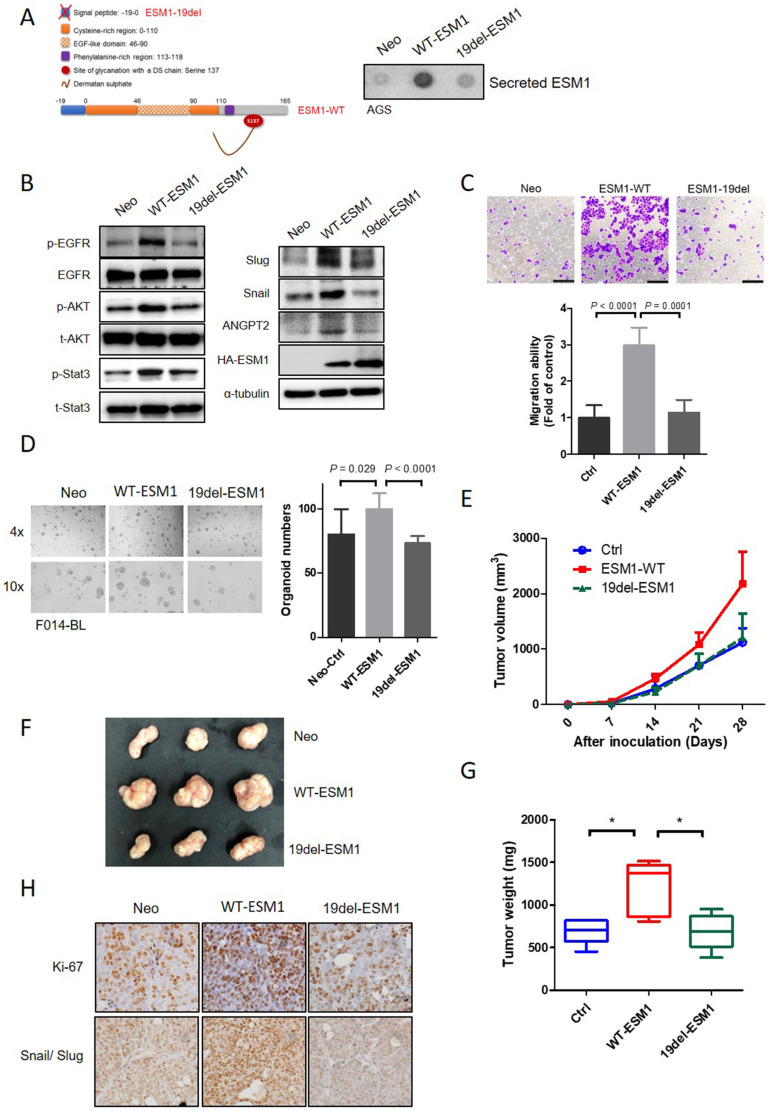
**Secretion of ESM1 is critical for ESM1-induced activation of the epidermal growth factor receptor (EGFR)-AKT axis to promote epithelial-to-mesenchymal transition (EMT)-mediated cell mobility in gastric cancer (GC) cells.** (**A, B**) Wild-type ESM1 (WT-ESM1) and 19del-ESM1 were introduced into AGS cells, and then cells were subjected to dot blot and Western blot assays to respectively detect the secretion of ESM1 (**A**) and activation of the EGFR-Akt-signal transduction and activator of transcription 3 (STAT3) axis or expressions of angiopoietin-2, Snail, and Slug (**B**). α-tubulin, EGFR, Akt, or STAT3 were used as an equal loading control. (**C, D**) AGS and patient-derived organoid (PDO) (F014-BL) cells were infected with a lentivirus-carrying control vector, WT-ESM1, or 19del-ESM1 as indicated. (**C**) Upper panel: Representative photos of transwell migration assays. Lower panel: Quantified migratory ability is presented as the mean ± SD. (**D**) The growth number of 3D PDOs was measured after carrying the control vector, WT-ESM1, or 19del-ESM1. (**E-G**) NCI-N87 cells carrying the control vector, WT-ESM1, or 19del-ESM1 were subcutaneously injected into mice, and the tumor growth curve (**E**), visualized tumor nodules (**F**), and tumor weight (**G**) were recorded as indicated. (**H**) NCI-N87 xenografts infected with the control vector, WT-ESM1, or 19del-ESM1 were isolated to detect expressions of Ki-67 and Snail/Slug by IHC staining. Original magnification, 400×.

Previous studies indicated that circulating levels of ESM1 increased over time and were positively correlated with GC tumor sizes 39, 40. Therefore, we constructed a nonsecreted mutant form of ESM1 by deleting a signal peptide, which involved loss the first 19 aas (ESM1-19del) (Figure 5A, left panel).

From results of a dot blot assay, secreted ESM1 was rarely detected in conditioned media (CM) from ESM1-19del-overexpressing AGS and NCI-N87 cells, but could be detected in CM from ESM1-wild type (WT)-overexpressing cells (Figures 5A, right panel, S5A). A Western blot analysis revealed lower levels of p-EGFR, p-Akt, p-STAT3, ANGPT2, Snail, and Slug in AGS and NCI-N87 cells overexpressing 19del-ESM1 compared to those overexpressing WT-ESM1 (Figures 5B and S5B). Functionally, AGS cells harboring the 19del-ESM1 mutant had a significantly lower migratory ability compared to cells harboring WT-ESM (Figure 5C). Notably, the tumor growth ability of F014-BL PDOs overexpressing 19del-ESM1 was also significantly lower than that of cells harboring WT-ESM1 (Figure 5D). The above observations indicated that ESM1 secretion was essential for ESM1-triggered activation of the EGFR-Akt-STAT3 axis and ANGPT2 expression to promote the EMT-mediated GC progression. In addition to the *in vitro* 2D and 3D culture systems, we further utilized an *in vivo* xenograft model to assess the importance of secreted ESM1 in facilitating cancer development. NCI-N87 cells overexpressing control vector (Ctrl), WT-ESM1, or 19del-ESM1 were subcutaneously injected into NOD-SCID mice, and sizes of tumor nodules were measured for 4 weeks. Mice bearing NCI-N87/WT-ESM1 exhibited larger tumor nodules compared to those implanted with NCI-N87/Ctrl and NCI-N87/19del-ESM1 (Figure 5E). Upon sacrifice of mice on the 28th day, visible tumor masses from implanted sites were collected and weighed. Data also revealed similar trends to measured tumor sizes (Figure 5F, G). To further investigate the necessity of secreted ESM1 for growth and EMT progression *in vivo*, we analyzed expression levels of proliferation and EMT markers using an IHC staining analysis. Consistent with our *in vitro* findings, we observed increased expression levels of Ki67 and both Snail/Slug in tumor tissues from mice injected with NCI-N87/WT-ESM1, compared to mice injected with NCI-N87/Ctrl or NCI-N87/19del-ESM1 (Figure 5H). These results all supported secreted ESM1 being necessary from GC growth and development.

### Targeting ESM1-drived EGFR/HER3 activation by therapeutic peptides inhibited the Akt-triggered EMT and motility of GC cells

**Figure 6:**
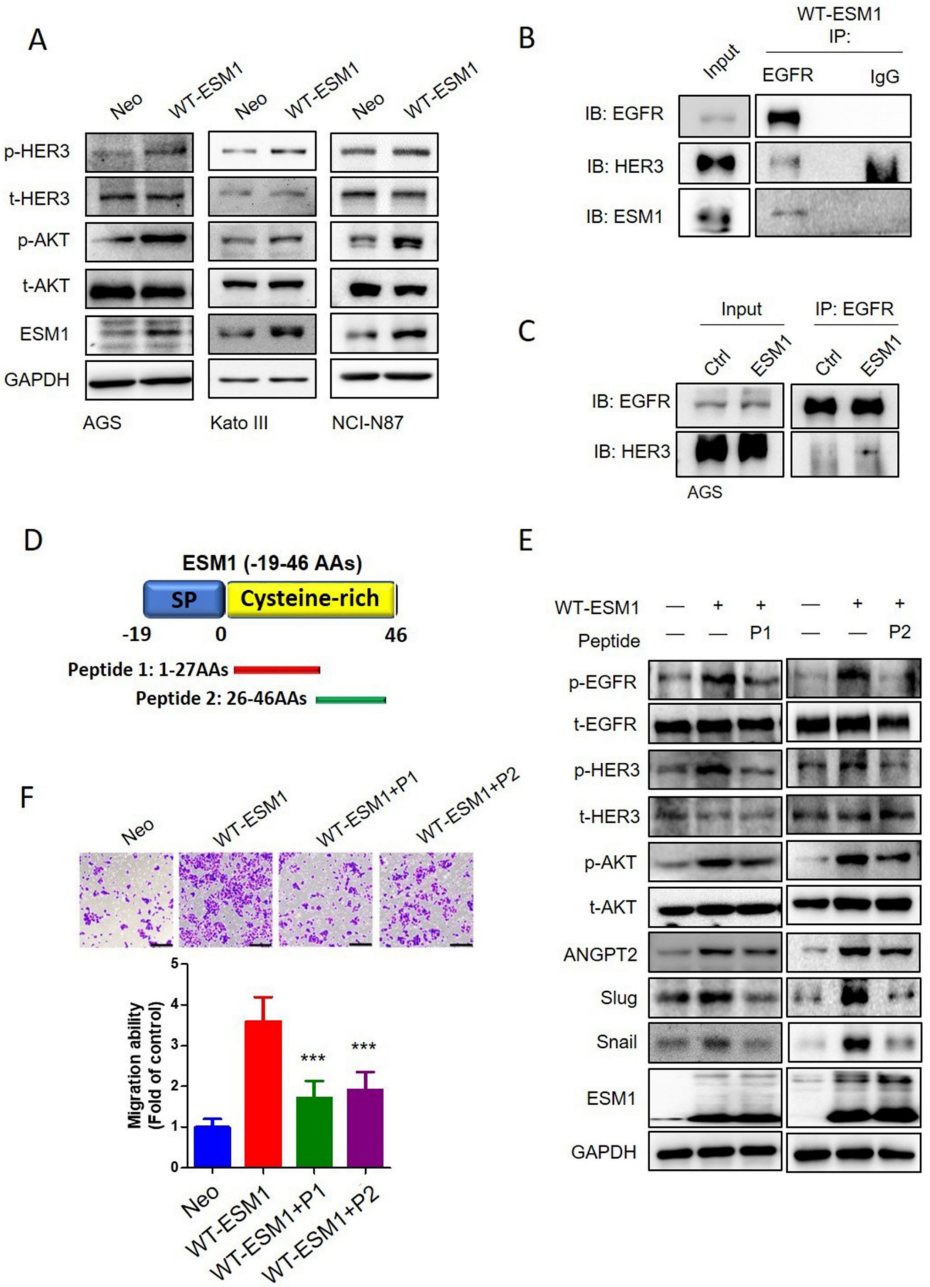
** Targeting the ESM1-epidermal growth factor receptor (EGFR) interaction by therapeutic peptides suppresses EGFR/human EGFR3 (HER3)-driven epithelial-mesenchymal transition (EMT) and cell mobility in gastric cancer (GC) cells.** (**A**) Wild-type ESM1 (WT-ESM1) was introduced into AGS, Kato-III, and NCI-N87 cells, and then cells were subjected to a Western blot assay to evaluate the phosphorylation status of HER3. (**B, C**) Co-immunoprecipitation assays were conducted to assess the interaction between EGFR, ESM1, and HER3 in AGS/WT-ESM cells (**B**). Comparing the association of EGFR and HER3 in the AGS cells transfected with either WT-ESM1 or a control vector (Ctrl) (**C**). Subsequently, a Western blot analysis was performed to examine the formation of this complex. (**D**) Schematic diagram of two synthetic peptides including peptide 1 (1-27 aas) and peptide 2 (26-46 aas) of the ESM1 protein. (**E, F**) AGS cells were infected with a lentivirus carrying a control vector or WT-ESM1 following treatment of cells with synthetic peptides (1 µM) as indicated. The phosphorylation status of HER3, EGFR and Akt, and expressions of angiopoietin-2, Snail, and Slug were all detected by Western blotting (**E**). The migratory ability of cells was determined by a transwell migration assay (**F**). Differences are presented as the mean ± SD. *** *p* < 0.001, compared to the WT-ESM1-overexpressing only group.

In addition to the EGFR, HER3 is another crucial member of the EGFR family that preferentially activates the PI3K/Akt pathway in various cancers 41, 42. High expression levels of HER3 in GC were often associated with poor outcomes 43. We observed that ESM1 overexpression also promoted HER3 activation in AGS, KATO-III, and NCI-N87 cells (Figure 6A), and ESM1 secretion was essential for ESM1-triggered HER3 activation (Figure S6A). We further observed the physical interaction of ESM1-EGFR-HER3 in AGS cells through co-immunoprecipitation experiments after pulling down EGFR (Figure 6B). Additionally, we found increased interactions between the EGFR and HER3 or p-HER3 in AGS/WT-ESM1 cells (Figures 6C, S6B). This indicated that the presence of ESM1 promoted the activation and formation of EGFR/HER3 complexes. In a previous study, we identified aas 1‒46 of ESM1 as crucial for the association with the extracellular domain of the EGFR and developed two peptide fragments of ESM1, including 1‒27 (peptide 1) and 26‒46 aas (peptide 2) (Figure 6D), to block the interaction of ESM1 and EGFR in lung cancer cells 17. Herein, we further evaluated the inhibitory effects of these peptides on EGFR-regulated signaling in GC cells. As shown in Fig. 6E, ESM1-triggered EGFR activation and its downstream signaling, such as Akt, ANGPT2, Snail, and Slug, were blocked by peptide 1 and peptide 2 treatment in AGS cells. Moreover, ESM1-induced HER3 activation was also suppressed by individual treatment with peptide 1 and peptide 2 in AGS cells (Figure 6E). Functionally, both peptides 1 and 2 showed significant inhibitory effects against the ESM1-induced increase in motility of AGS cells (Figure 6F). These results suggest the potential clinical application of synthetic ESM1 peptides in GC therapy by blocking activation of the EGFR/HER3-Akt axis.

## Discussion

The late onset of symptoms, high recurrence rates, and poor survival rates have made GC the fourth leading cause of cancer-related deaths worldwide 1. There is an urgent need to identify novel diagnostic and prognostic biomarkers and therapeutic targets, and investigate potential molecular mechanisms of GC. In the clinic, we found that ESM1 was upregulated in GC and was associated with advanced clinical stages, lymph node metastases, vascular invasion, and poor prognoses. Elevated ESM1 levels could serve as an independent prognostic factor in GC patients.

*In vitro*, ESM1-KD inhibited the growth or motility of GC cells and PDOs by suppressing proliferation, colony formation, anoikis resistance, migration, and invasion, while ESM1 overexpression had the opposite effects. The EMT, a critical step in the initiation and promotion of tumor proliferation, migration, and metastasis in GC cells 7, was also crucial for ESM1-modulated progression. Our findings, consistent with previous studies, indicated that ESM1 could induce the EMT to promote the progression of colorectal carcinoma 18. Mechanistically, we identified that ESM1 induces the association and activation of the EGFR and HER3 to activate Akt/ANGPT2 signals, subsequently triggering the EMT. Additionally, we demonstrated that ESM1 secretion was crucial for its oncogenic role in GC cells, PDOs, and GC xenograft models. Finally, targeting ESM1 with therapeutic peptides successfully reduced EGFR/HER3 signaling and alleviated the motility of GC cells.

The EMT is known to be regulated by signals from the tumor microenvironment, including various cytokines and growth factors such as EGF, hepatocyte growth factor (HGF), tumor necrosis factor-α (TNF-α), and TGF-β, which can activate several transcription factors promoting the EMT, including NF-κB, Snail, Slug, ZEB1, and Twist, to transmit EMT promotion signals 44, 45. The enrichment of TGF-β and TNF-α/NF-κB signal in GC patients with ESM1^high^ was also observed (Fig 3F). Our GSEA analysis from GC patients echoes the cyclic regulation loop of TNF-α/NF-κB and ESM1 signaling which was reported in previous studies. For example, the activation of TNF-α upregulated ESM1 expression by the RelB subunit of NF-κB in breast cancer cells 46. On the other hand, ESM1 was shown to upregulate the NF-κB in human umbilical vein endothelial cells 47. The cross-talk of ESM1 and TNF-α/NF-κB signaling in GC progression is also worthy of further investigation. Our previous study showed that ESM1 could directly bind to the extracellular domain (ECD) of the EGFR and enhance EGF binding, thereby increasing EGFR phosphorylation and downstream signal transduction in lung cancer 17. EGFR, overexpressed in 27%-64% of GC cases, is well-known for its oncogenic role in this malignancy 41. Herein, we observed that ESM1 overexpression increased EGFR phosphorylation and its downstream signals, Akt and STAT3, in AGS, NCI-N87, and KATO-III cells. Moreover, we found that the Akt-specific inhibitor, MK-2206, significantly reversed ESM1 overexpression-induced activation of Akt and STAT3, as well as Snail and Slug upregulation. Functionally, MK-2206 treatment dominantly rescued ESM1-induced increases in motility and anoikis resistance, suggesting that ESM1 induces GC progression through triggering the EMT, by involving activation of the EGFR-Akt-STAT3 signaling pathway. In lung cancer, activation of the EGFR-AKT-STAT3 pathway was reported to influence antitumor immune responses by regulating programmed death ligand 1 (PD-L1) expression 48. PD-L1 was reported to be elevated in up to 50%-60% of GC cases 49. Whether ESM1 can affect PD-L1 expression and antitumor immune responses in GC should be further investigated. Cetuximab, a monoclonal antibody that binds to the ECD of EGFR, competitively inhibits EGF binding and blocking of ligand-induced EGFR activation 50. EGFR activation leading to the EMT was associated with a metastatic phenotype and reduced sensitivity of head and neck squamous cell carcinoma (HNSCC) cells to cetuximab treatment 51. Therefore, we suggest that ESM1 might reduce the response to cetuximab treatment in GC through the EGFR activation-triggered EMT, and this possibility needs to be further investigated in the future.

In addition to the EGFR, HER3 overexpression is frequently observed in GC 41 and was shown to interact with the EGFR or HER2 to form a heterodimeric complex in the presence of its own ligand, neuregulin. In contrast, when EGFR homomeric complexes preferentially form in the presence of EGF, HER3 does not form asymmetric kinase dimers with the EGFR, but forms clusters to promote its phosphorylation 52. Activation of HER3 dominantly activates the phosphatidylinositol 3-kinase (PI-3K)/Akt pathway, ultimately inducing the EMT and cancer progression 41, 53. Surprisingly, we observed that ESM1 overexpression enhanced an association of the EGFR and HER3 and induced HER3 phosphorylation in GC cells. Moreover, blocking the ESM1-EGFR interaction with our synthetic ESM1 peptides 17 not only suppressed ESM1-triggered EGFR phosphorylation but also HER3 phosphorylation and their downstream oncogenic signals, including ANGPT2, Akt, Snail, and Slug. Furthermore, these therapeutic peptides significantly reversed the ESM1 overexpression-induced increase in motility of GC cells. Collectively, our results suggest that ESM1 may promote GC progression by activating the EGFR/HER3 complex. Recent reports indicated that cetuximab treatment induces HER3 activation in cancers 54, 55, and dual targeting of the EGFR and HER3 can overcome acquired resistance to cetuximab or the EGFR tyrosine kinase inhibitor (TKI), erlotinib, further highlighting the role of HER3 in EGFR-targeted therapy 55, 56. Treatment with our ESM1 peptides reduced phosphorylation levels of both the EGFR and HER3, suggesting the potential clinical application of these synthetic ESM1 peptides to treat cetuximab-resistant patients. In addition to HER3, a survival analysis using a KM plotter indicated that ESM1 has significant prognostic value in HER2^+^ patients but not in HER2^-^ patients (Figure S7). These findings suggest potential regulatory roles of ESM1 in HER2, another critical receptor tyrosine kinase in the ERBB family, warranting further investigation.

To further dissect the targeted regulation of ESM1, we found that ANGPT2 was most strongly correlated with ESM1 in GC samples, and ESM1 overexpression induced upregulation of ANGPT2 in AGS and KATO-III cells. This positive correlation phenomenon was also observed in HNSCC 57. Several crucial transcription factors, including STAT3, HOXD9, and HOXB5, are known to bind to ANGPT2 promoter regions, thereby activating ANGPT2 expression and promoting malignant phenotypes in cancer cells 58-60. Moreover, EGFR, TGF-β, and PI3K/AKT/HIF1α signals serve as upstream activators for STAT3 61, HOXD9 62, and HOXB5 63. Our results revealed the ESM1-involved activation of EGFR, TGF-β, and AKT signals. We believe that these oncogenic pathways likely play roles in ESM1-induced ANGPT2 expression and warrant further investigation. Accumulating evidence indicated that ANGPT2 expression by tumor cells or elevated circulating ANGPT2 is closely linked with invasive and metastatic phenotypes of various cancer types including GC 64. In GC cells, ANGPT2 was shown to encourage growth, invasion, and EMT progression 65, and similar phenomena were also observed in lung 66 and breast 67 cancers. Our present study showed that ANGPT2-KD significantly reversed ESM1-induced activation of Akt, upregulation of mesenchymal markers, and increased migratory and growth abilities of GC cells. In the clinic, we observed that ANGPT2 expression in GC samples was positively correlated with expressions of mesenchymal markers. These results suggested that ANGPT2 may contribute to ESM1-trigged activation of the EGFR-Akt axis, EMT, and progression of GC cells. Taken together, our results suggested crosstalk between ANGPT2 and Akt signaling involved in ESM1-promoted progression of GC. However, the complex interplay between ANGPT2 and Akt signaling in GC needs to be further investigated.

## Conclusions

**Figure 7:**
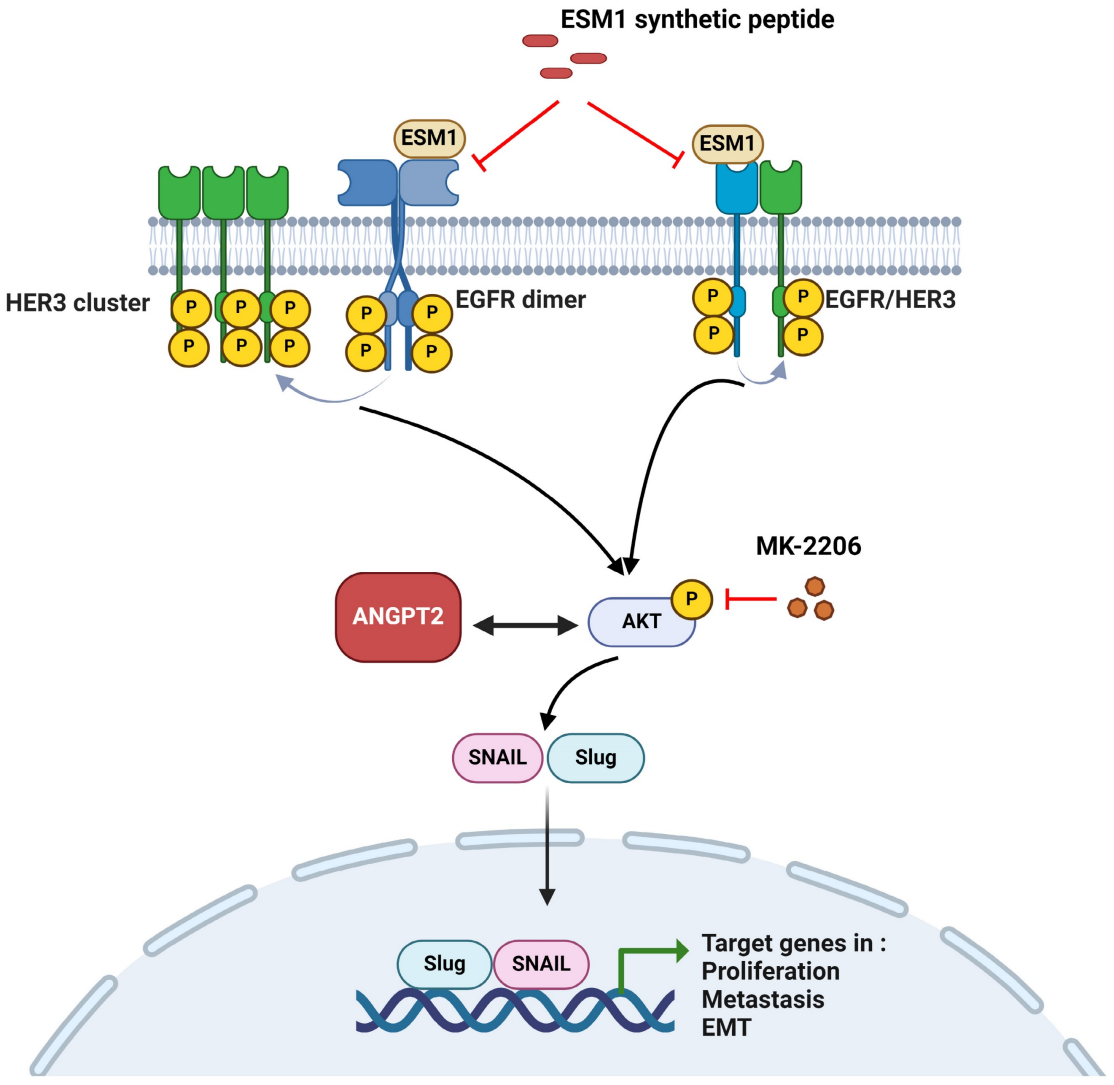
** A working model shows the molecular mechanism underlying the ability of ESM1 to promote progression of gastric cancer (GC) cells.** The oncogenic role of ESM1 was attributed to triggering the epithelial-to-mesenchymal transition (EMT) by activating epidermal growth factor receptor (EGFR)/human EGFR3 (HER3) and their downstream signal, Akt. Angiopoietin-2 was highly correlated with ESM1 and interplayed with Akt to promote EMT progression. Blocking the interaction of ESM1 and the EGFR by synthetic ESM1 peptides attenuated the EGFR/HER3 activation-driven EMT, cell motility, and proliferation. This schematic representation was created using BioRender software.

In summary, we present evidence showing that ESM1 plays a crucial role in GC progression. ESM1 promotes GC growth and metastasis abilities via activating the EGFR/HER3-Akt/ANGPT2 pathway and promoting EMT progression. Meanwhile, we also demonstrated that secretion of ESM1 was critical for the aforementioned ESM1-modulated signal pathways to promote GC progression. The mechanism is schematically illustrated in Figure 7. Our findings indicated that ESM1 could be an effective biomarker and novel therapeutic target for patients with GC.

## Supplementary Material

Supplementary figures and tables.

## Abbreviations

aas: amino acids
ANGPT2: angiopoietin-2
CM: conditioned medium
DFS: disease-free survival
EGFR: epidermal growth factor receptor
EMT: epithelial-to-mesenchymal transition
ESM1: endothelial cell-specific molecule 1
GC: gastric cancer
GEO: Gene Expression Omnibus
IHC: immunohistochemistry
HER3: human epidermal growth factor receptor 3
IF: immunofluorescence
NOD: nonobese diabetic
OS: overall survival
PDO: patient-derived organoid
RT-PCR: reverse-transcriptase polymerase chain reaction
TCGA: The Cancer Genome Atlas
WT: wild type
