# Supplementary material for: ESM1 facilitates the EGFR/HER3-triggered epithelial-to-mesenchymal transition and progression of gastric cancer via modulating interplay between Akt and angiopoietin-2 signaling
Source: Int J Biol Sci. 2024 Sep 9;20(12):4819–37. doi: 10.7150/ijbs.100276 (PMC11414391; doi:10.7150/ijbs.100276)
Supplement: Supplementary file 1 — Supplementary figures and tables. [file ijbsv20p4819s1.pdf]

Supplementary information

Title:

ESM1 facilitates the EGFR/HER3-triggered epithelial-to-mesenchymal transition and progression of gastric cancer via modulating interplay between Akt and angiopoietin-2 signaling

Yi-Chieh Yang, Ko-Hao Ho, Ke-Fan Pan, Kuo-Tai Hua, Min-Che Tung, Chia-Chi Ku, Ji-Qing Chen, Michael Hsiao, Chi-Long Chen, Wei-Jiunn Lee, Ming-Hsien Chien (E-mail: mhchien1976@gmail.com)

Table S1 Correlation of clinical features and ESM1 expression.

Clinicopathological feature	N<br>300	ESM1 expression, n (%)<br>Low<br>200 (66.7)	 High<br>100 (33.3)	*P*
Age				
<65 y	161	106	55	0.743
≧65 y	139	94	45	
Gender				
Male	199	123	76	***0.012***
Female	101	77	24	
Lauren's Classification				
Intestinal type	146	97	49	0.985
Diffuse type	135	90	45	
Mixed type	19	13	6	
Stage				
I+II	127	90	37	0.556
III+IV	173	110	63	
T stage				
T1+T2	188	123	65	0.555
T3+T4	112	77	35	
N stage				
N0+N1	169	123	46	***0.011***
N2+N3	131	77	54	
M stage				

M0	273	186	87	0.087
M1	27	14	13	
Perineural Invasion				
No	159	105	54	0.872
Yes	88	59	29	
Not reported	53	36	17	
Venous Invasion				
No	129	89	40	***0.023***
Yes	44	22	22	
Not reported	127	89	38	
Lymphatic invasion				
No	73	55	18	0.087
Yes	205	132	73	
Not reported	22	13	9	

Table S2. Univariate and multivariate analysis including ESM1 expression and various clinicopathological parameters on overall survival

Variables		OS	
		HR (95% CI)	*P*
Cox univariate analysis			
Gender	Male vs. Female	0.905 (0.65-1.27)	0.559
Age	≧65 vs. <65	1.55 (1.13-2.13)	0.007
ESM1	High vs. low	1.52 (1.10-2.10)	0.012
T stage	T3-4 vs. T1-2	2.395 (1.74-3.3)	<0.001
N stage	N23 vs. N10	2.78 (2.00-3.85)	<0.001
M stage	M1 vs. M0	3.84 (2.48-5.94)	<0.001
Cox multivariate analysis			
Age	≧65 vs. <65	1.78 (1.29-2.46)	<0.001
ESM1	High vs. low	1.43 (1.03-2.00)	0.034
T stage	T3-4 vs. T1-2	2.08 (1.49-2.91)	<0.001
N stage	N1-2 vs. N0	2.06 (1.45-2.94)	<0.001
M stage	M1 vs. M0	2.52 (1.61-3.95)	<0.001

OS: overall survival; DFS: disease-free survival; HR: hazard ratio; CI: confidence interval

Figure Legends

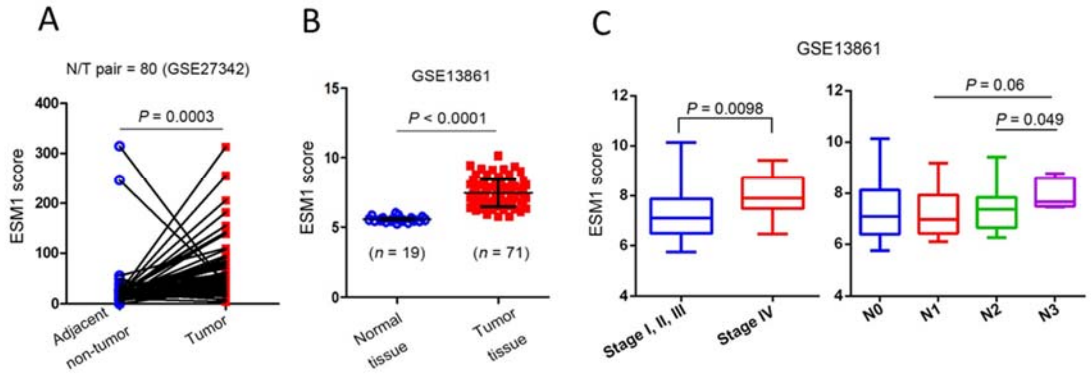

Figure S1. *In silico* analysis of gastric cancer samples from the GSE27342 and GSE13861 datasets. A, B Expression of ESM1 in normal and gastric cancer tissues. **C** Expression of ESM1 in gastric cancer with different clinical and lymph node metastasis statuses.

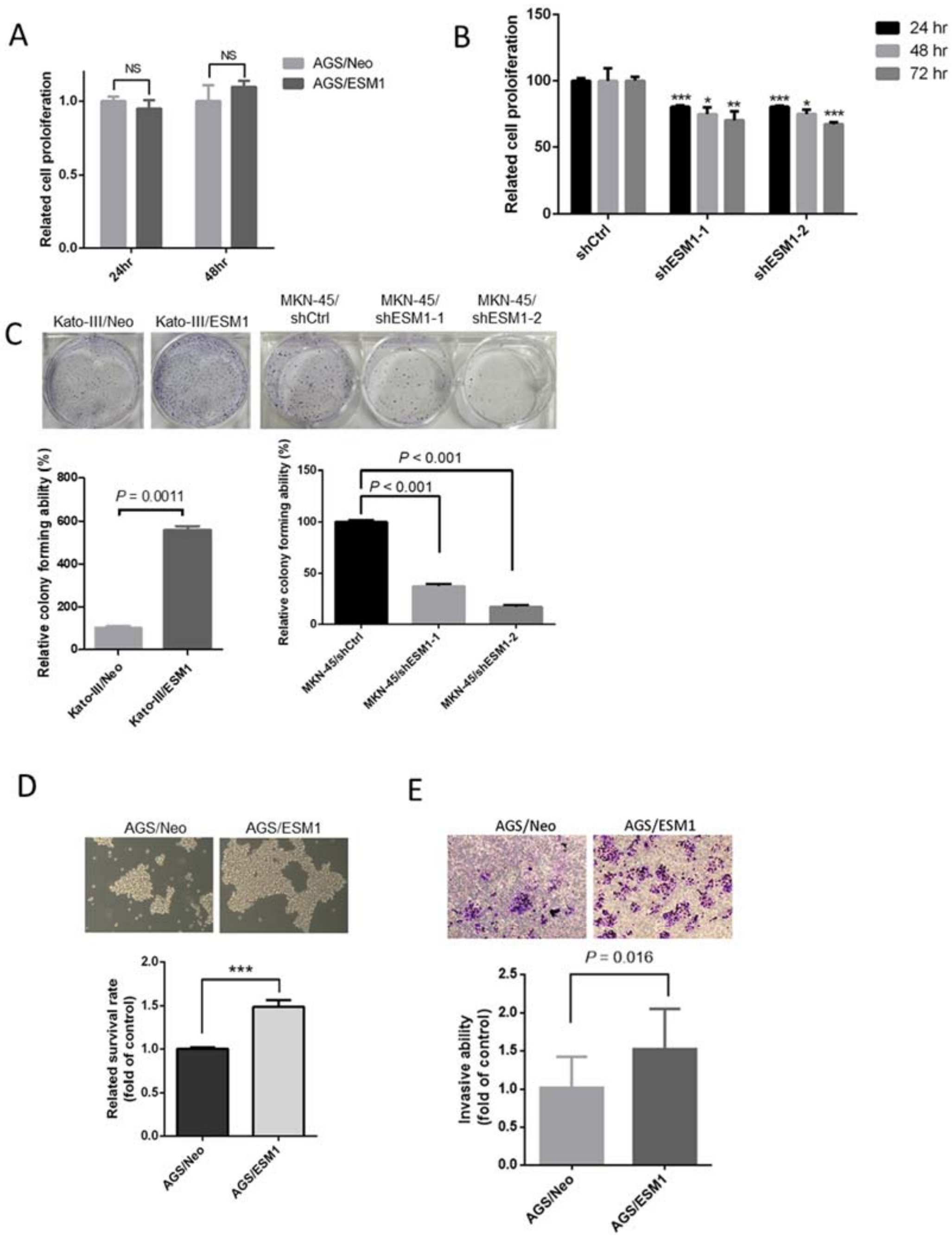

Figure S2. Manipulation of ESM1 affects cancer cell behaviors. A, B The cell proliferation ability of AGS cells with overexpression and knockdown of ESM1. **C** Overexpression and knockdown of ESM1 respectively promoted and attenuated the colony-forming ability of KATO-III and MKN-45 cells. **D, E** Anoikis resistance and invasion assays of the empty vector-control *vs*. ESM1-overexpressing AGS cells. *** $p<0.001$ versus the control group.

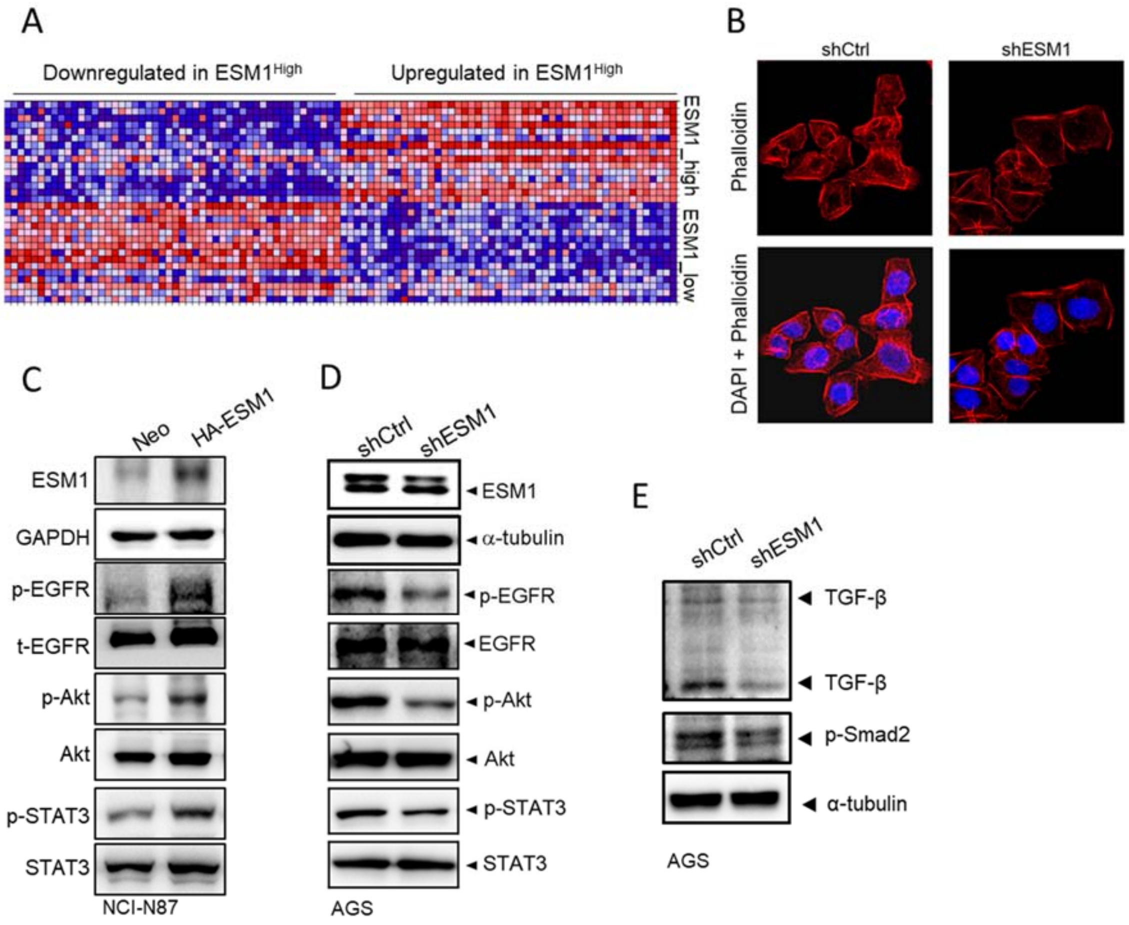

Figure S3. ESM1 promotes the epithelial-to-mesenchymal transition (EMT) of gastric cancer and EGFR-related downstream signals, Akt, STAT3, and TGF-β. **A** Heatmap of the 50 highest distinguished gene clusters for ESM1 high/low genotype in GC patients. **B** Cellular microfilament bundle rearrangements were induced by knocking down ESM1 in AGS cells. Cells were fixed and stained for F-actin by Alexa Fluor 594 phalloidin. Nuclei were counterstained with DAPI (blue). **C-E** Western blot analysis to examine the EGFR and relative downstream signals in ESM1-overexpressing NCI-N87 (**C**) and ESM1-knockdown **(D, E)** AGS cells.

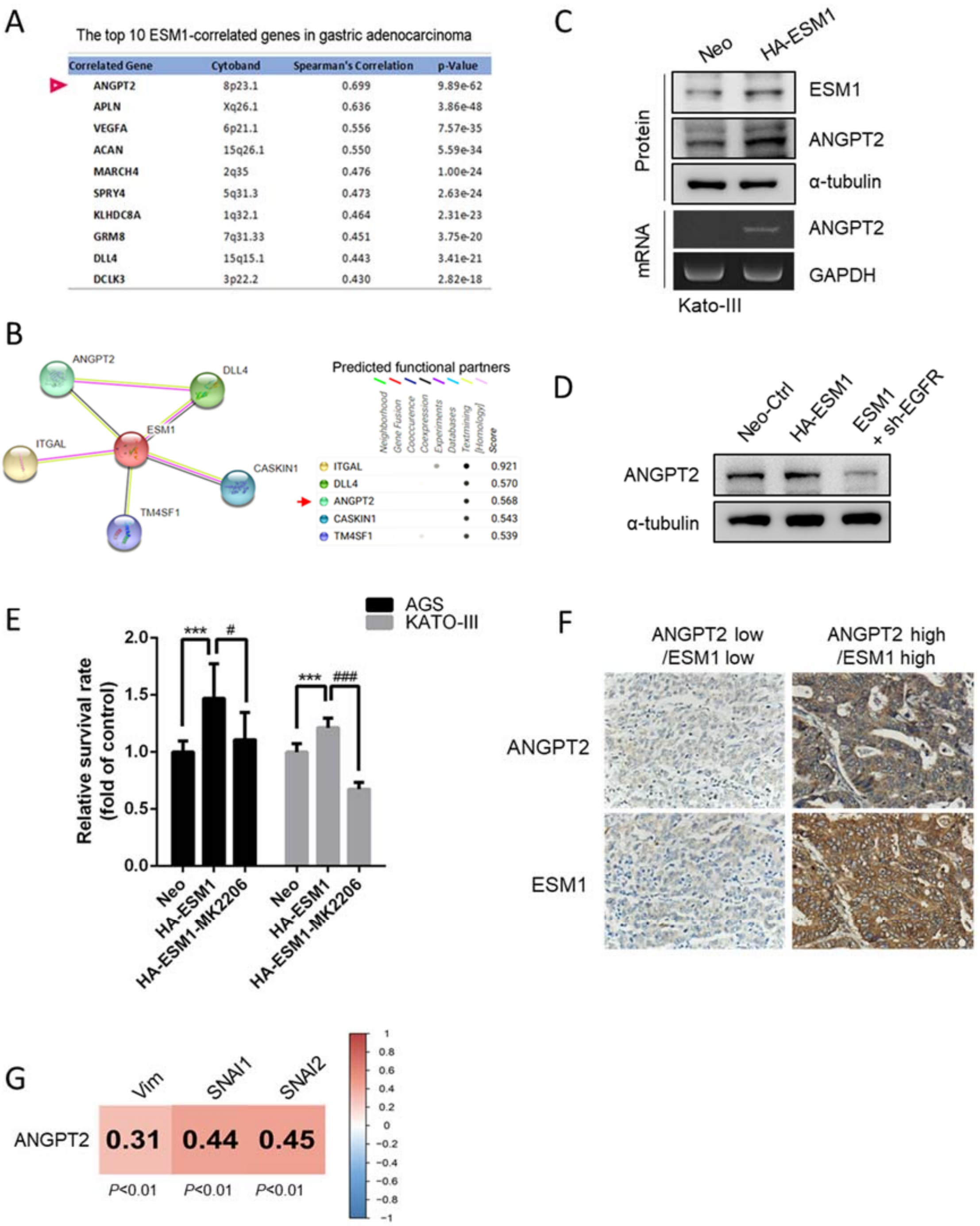
A
The top 10 ESM1-correlated genes in gastric adenocarcinoma
Correlated Gene
Cytoband
Spearman's Correlation
p-Value
ANGPT2 8p23.1 0.699 9.89e-62
APLN Xq26.1 0.636 3.86e-48
VEGFA 6p21.1 0.556 7.57e-35
ACAN 15q26.1 0.550 5.59e-34
MARCH4 2q35 0.476 1.00e-24
SPRY4 5q31.3 0.473 2.63e-24
KLHDC8A 1q32.1 0.464 2.31e-23
GRM8 7q31.33 0.451 3.75e-20
DLL4 15q15.1 0.443 3.41e-21
DCLK3 3p22.2 0.430 2.82e-18
B
ANGPT2
DLL4
ITGAL
ESM1
CASKIN1
TM4SF1
Predicted functional partners
Neighborhood
Gene Fusion
Cooccurence
Coexpression
Experiments
Databases
Textmining
[Homology]
Score
ITGAL 0.921
DLL4 0.570
ANGPT2 0.568
CASKIN1 0.543
TM4SF1 0.539
C
Neo
HA-ESM1
Protein
ESM1
ANGPT2
α-tubulin
mRNA
ANGPT2
GAPDH
Kato-III
D
Neo-Ctrl
HA-ESM1
ESM1 + sh-EGFR
ANGPT2
α-tubulin
E
AGS
KATO-III
Relative survival rate (fold of control)
2.0
1.5
1.0
0.5
0.0
***
#
***
###
Neo
HA-ESM1
HA-ESM1-MK2206
Neo
HA-ESM1
HA-ESM1-MK2206
F
ANGPT2 low /ESM1 low
ANGPT2 high /ESM1 high
ANGPT2
ESM1
G
Vim
SNAI1
SNAI2
ANGPT2
0.31
0.44
0.45
P<0.01
P<0.01
P<0.01
1
0.8
0.6
0.4
0.2
0
−0.2
−0.4
−0.6
−0.8
−1

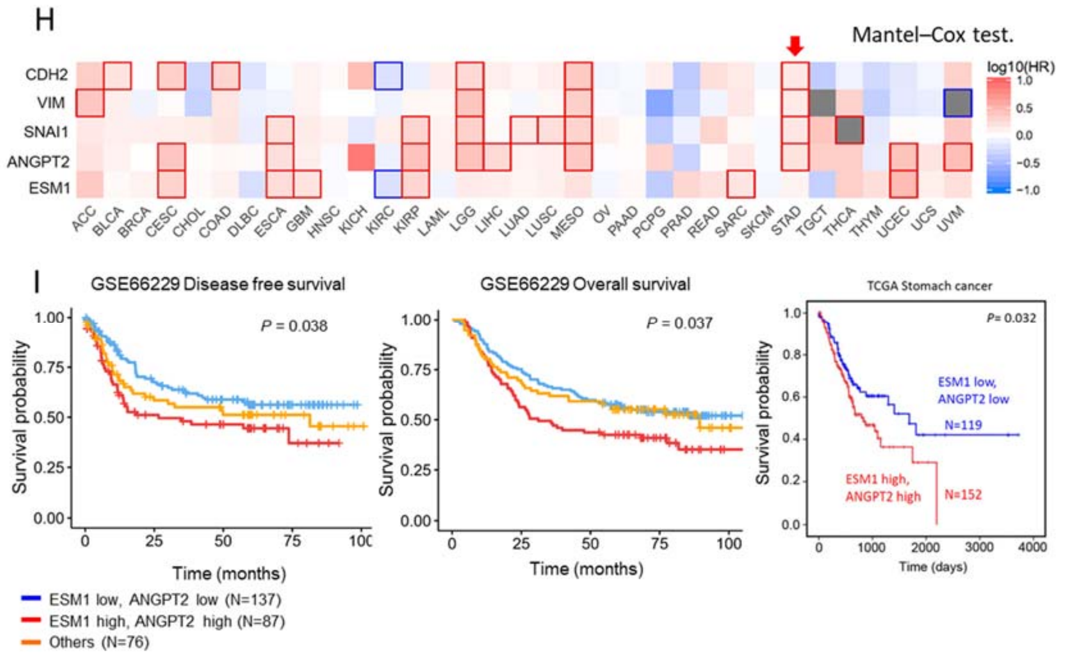

Figure S4. Expression of angiopoietin-2 (ANGPT2) was positively correlated with ESM1 and cooperated with its oncogenic properties. **A** List of the top 10 ESM1-correlated genes with the highest Spearman's correlation coefficient obtained from the cBioPortal database. **B** The protein-protein interaction network of ESM1 and five candidate targets from the STRING database. **C** mRNA and protein levels of ANGPT2 in ESM1-overexpressing Kato-III cells. **D** Western blot analysis examining the level of ANGPT2 under manipulating ESM1 and EGFR levels in AGS cells. **E** Anoikis resistance of the ESM1-overexpressing versus vector-control or ESM1-overexpressing combined with MK-2206 treatment in AGS and Kato-III cells. *** p<0.001 ESM1 versus the control group; $^{\#}$ p<0.05, $^{\#\#\#}$ p<0.0001 ESM1 versus the ESM1+MK2206 group. **F** Representative pictures of IHC staining of ESM1 and ANGPT2 level in gastric cancer patients. **G** Positive correlations of ANGPT2 and mesenchymal markers of vimentin (VIM), Snail (SNAI1), and Slug (SNAI2) in GC patients. **H** Visualizing a survival map and hazard ratio of CDH2, VIM, SANI1, SNAI2, ANGPT2, and ESM1 expression statuses in different cancer types. **I** Kaplan-Meier curves for overall GC

patient survival, grouped by ESM1 and ANGPT2 expression. The *p* value indicates a comparison between patients with $ESM1^{high}/ANGPT2^{high}$, those with $ESM1^{low}/ANGPT2^{low}$, and others. The GC dataset was retrieved from GSE66229 and TCGA.

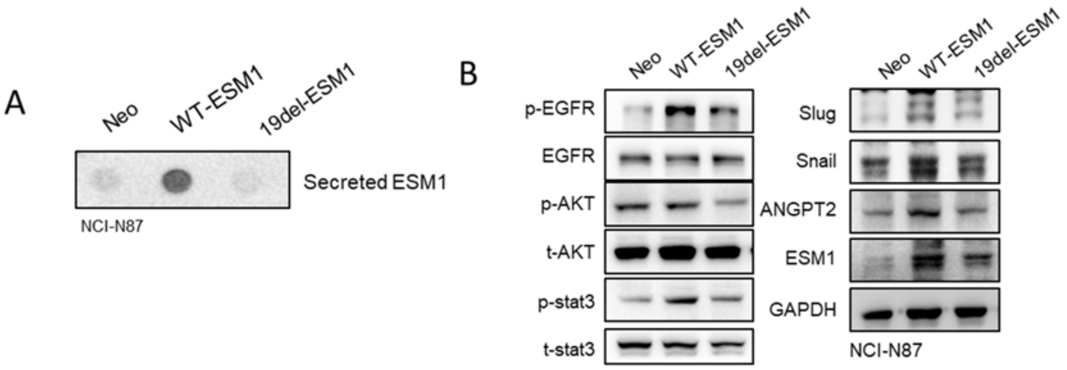

Figure S5. Secreted ESM1 enhances activation of the epidermal growth factor receptor (EGFR) and related downstream signals. A, B Wild-type ESM1 (WT-ESM1) and 19del-ESM1 were introduced into NCI-N87 cells, and then cells were subjected to dot blot and Western blot assays to respectively detect the secretion of ESM1 (**A**) and activation of the EGFR-Akt-STAT3 axis or expressions of angiopoietin-2, Snail, and Slug (**B**).

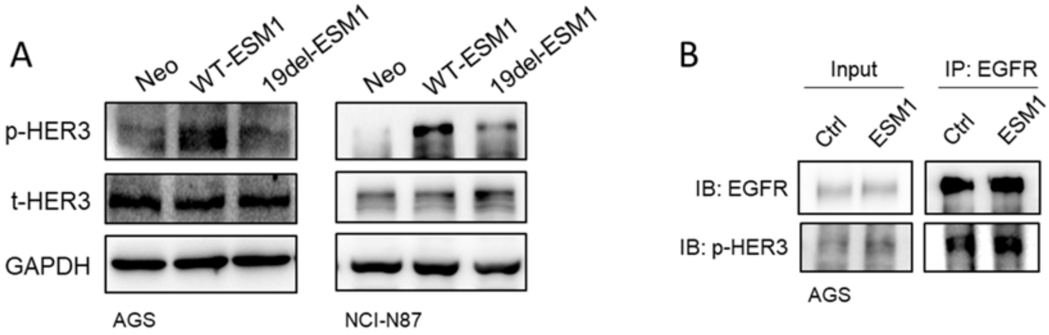

Figure S6. Activation of human epidermal growth factor receptor 3 (HER3) depends on the secreted ESM1. A WT-ESM1 and 19del-ESM1 were introduced into AGS and NCI-N87 cells, and then cells were subjected to Western blot assays to detect activation of HER3. **B** Co-immunoprecipitation assays were conducted to assess the interaction between the EGFR and phosphorylated (p)-HER3 in AGS cells.

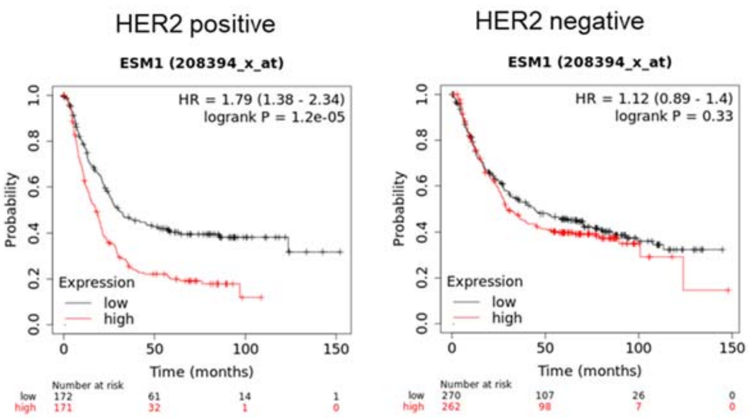

Figure S7. High ESM1 expression predicted a poor prognosis in HER2 positive gastric cancer (GC) patients, but not in HER2 negative patients. Correlation between ESM1 expression and survival outcomes in gastric cancer (GC) patients relying on different HER2 statuses which were retrieved from a KM plotter database. Gene expression was dichotomized into high and low values using the median as a cutoff.
